# Attenuation of sensory processing in the primary somatosensory cortex during rubber hand illusion

**DOI:** 10.1038/s41598-021-86828-5

**Published:** 2021-04-01

**Authors:** Masanori Sakamoto, Hirotoshi Ifuku

**Affiliations:** Department of Physical Education, Faculty of Education, Kumamoto University, 2-40-1 Kurokami, Chuo-ku, Kumamoto, 860-8555 Japan

**Keywords:** Cognitive neuroscience, Sensory processing, Somatosensory system

## Abstract

The neural representation of the body is easily altered by the integration of multiple sensory signals in the brain. The “rubber hand illusion” (RHI) is one of the most popular experimental paradigms to investigate this phenomenon. During this illusion, a feeling of ownership of the rubber hand is created. Some studies have shown that somatosensory processing in the brain is attenuated when RHI occurs. However, it is unknown where attenuation of somatosensory processing occurs. Here, we show that somatosensory processing is attenuated in the primary somatosensory cortex. We found that the earliest response of somatosensory evoked potentials, which is thought to originate from the primary somatosensory cortex, was attenuated during RHI. Furthermore, this attenuation was observed before the occurrence of the illusion. Our results suggest that attenuation of sensory processing in the primary somatosensory cortex is one of the factors influencing the occurrence of the RHI.

## Introduction

Multiple sensory signals are integrated in the brain, which contribute to shaping the neural representation of the body^1,2^, which easily altered under certain circumstances. One of the most popular experimental paradigms for investigating this phenomenon is the ‘rubber hand illusion’ (RHI)^3^. In the paradigm, watching a fake rubber hand being stroked by a paintbrush in synchrony and in the same direction with one’s own concealed hand creates the feeling that the rubber hand is one’s own. Therefore, when visual and tactile signals are integrated into the brain, a feeling of ownership of the rubber hand is created^3–6^. The posterior parietal cortex is likely to be involved in multisensory integration during RHI^4,7–11^.

Although many previous studies have demonstrated that the ventral premotor and posterior parietal cortices are related to the occurrence of the RHI^4,7–11^, the role of the primary somatosensory cortex is controversial. Rao and Kayser^12^ used electroencephalography (EEG) and demonstrated that neurophysiological correlates of the RHI were observed in the frontocentral areas. Guterstam et al.^11^ showed RHI-related activities in the premotor and intraparietal cortices using electrocorticography (ECoG) in patients. However, this study did not show modulation of responses in the primary somatosensory cortex. These findings are in line with earlier neuroimaging studies showing neural correlates of RHI in the premotor and parietal cortices^4,7–11^. In contrast to these studies, modulations of somatosensory processing in the primary somatosensory cortex during RHI have been reported using a variety of methods, including somatosensory evoked potentials^13^, transcranial magnetic stimulation^14^, and multi-unit neural recording in monkeys^15^. Although the cause of the inconsistent results seems to be the difference in the methodology used, clarifying the contribution of the primary somatosensory cortex is important for elucidating the neural mechanisms of the RHI.

During the RHI, participants erroneously perceive the fake hand as their own, that is, they fail to perceive their real hand as their own. In this case, the question is how somatosensory signals from their real hand induced by tactile stimulation are transmitted to the brain. Previous studies have investigated the matter^13,16–18^. Zeller et al.^13^ recorded somatosensory evoked potentials (SEPs) elicited by a brush stroke during RHI, and a response of approximately 50 ms after stroking was attenuated. The component was thought to originate from the primary somatosensory cortex, although it did not reflect the initial stage of sensory processing in the area^19,20^. This suggested that the relative attenuation of somatosensory processing might be accompanied by the occurrence of RHI^21^.

We consider two critical problems that remain open. First, we investigated whether attenuation of somatosensory processing occurs even in the earliest stage of information processing in the primary somatosensory cortex. Second, whether the occurrence of the RHI is followed by modulation of somatosensory processing in the primary somatosensory cortex, or the modulation of somatosensory processing occurs before the occurrence of the illusion. To address the first issue, we recorded SEPs elicited by electrical stimulation of the peripheral nerve instead of the brush^13,16–18^, or vibration^12^-evoked potentials used in multiple previous studies. This is because the brush- or vibration-evoked potentials do not recognise the earliest response of the primary somatosensory cortex that is observed approximately 20 ms after stimulation^22–25^. The earliest component is generated from Brodmann’s area 3b^26,27^ and is thought to reflect the initial sensory processing in the primary somatosensory cortex. To elucidate the second matter, we asked the participants to report the timing of the occurrence of the RHI. This allowed us to record SEPs before and after the occurrence of the illusion. This study was designed to elucidate the above two questions and to understand the neural mechanisms underlying the occurrence of RHI.

## Results

### Experiment 1

**Figure 1:**
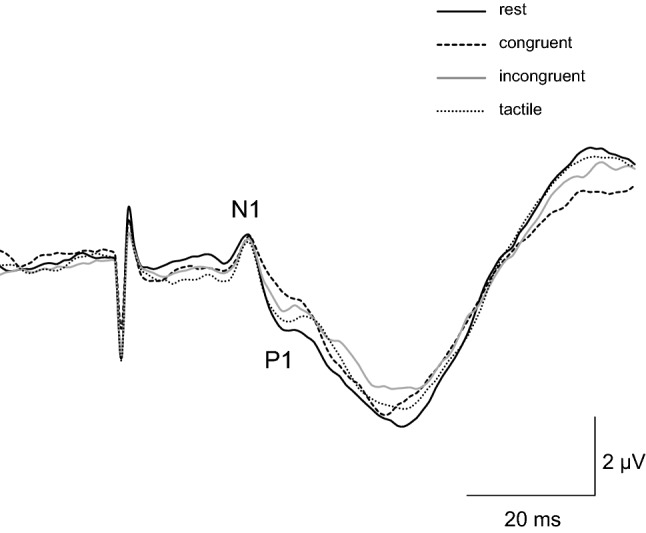
Grand averaged waveforms of somatosensory evoked potentials (SEPs) during all conditions in Experiment 1. The first negative peak approximately 20 ms after electrical stimulation (N1) and subsequent positive peak at about 25 ms (P1) were clearly identified in all conditions.

Figure 1 shows the grand averaged waveforms of the SEP in all the conditions. The N1-P1 component was consistently recorded in all participants. It can be seen that the component showed an attenuation in amplitude in the congruent stroking condition. The number of averages SEPs in the rest, congruent stroking, incongruent stroking, and tactile stimulation conditions were 37.2 ± 4.6, 34.8 ± 5.1, 33.1 ± 4.2, and 37.1 ± 4.4, respectively. The difference in the number of averages was due to the exclusion of trials with participants’ blinking owing to SEP averaging.

**Figure 2:**
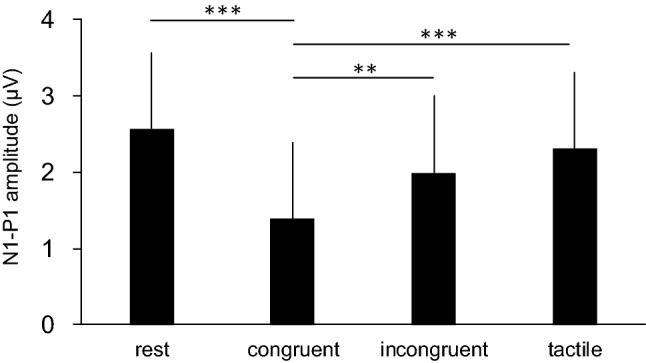
Group means of N1-P1 amplitudes during the rest, and the congruent, incongruent, and tactile stroking conditions in experiment 1. Data are represented as mean ± one SD. **p < 0.01, ***p < 0.001.

The amplitudes of the N1-P1 components in all the conditions are shown in Fig. 2. One-way repeated measures ANOVA revealed a significant main effect for the conditions (F(1.9,28.7) = 18.25, *p* = 0.0001, η^2^ = 0.55). Post-hoc comparisons demonstrated that the N1-P1 component in the congruent stroking condition was significantly smaller than that in the rest condition (*p* = 0.00004), incongruent stroking (*p* = 0.008), and tactile stimulation condition (*p* = 0.0002). The N1-P1 amplitude in the tactile stimulation condition had a tendency to decrease compared to that in the rest condition(*p* = 0.055).

**Figure 3:**
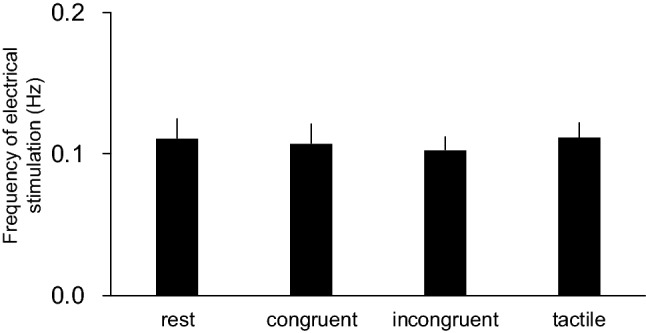
Group means of frequency of electrical stimulation during the rest, congruent, incongruent, and tactile stroking conditions in Experiment 1.

Figure 3 demonstrates the frequency of electrical stimulation across all conditions. The interstimulus interval in all the conditions was approximately 10 s. One-way repeated measures ANOVA revealed no significant main effect for the conditions (F(3,45) = 2.1, *p* = 0.11, η^2^ = 0.12).

**Figure 4:**
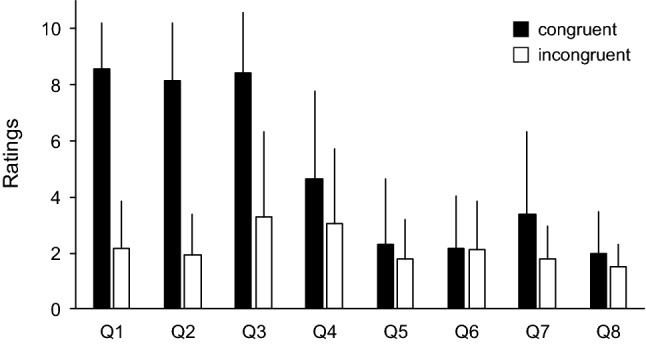
Questionnaire data showing the mean ratings in the congruent (black columns) and incongruent (white columns) stroking condition in Experiment 1. Ratings for the questionnaire statements on a 10-point scale ranging from 1 to 10, with 1 corresponding to *strongly disagree* and 10 to *strongly agree.*

**Figure 5:**
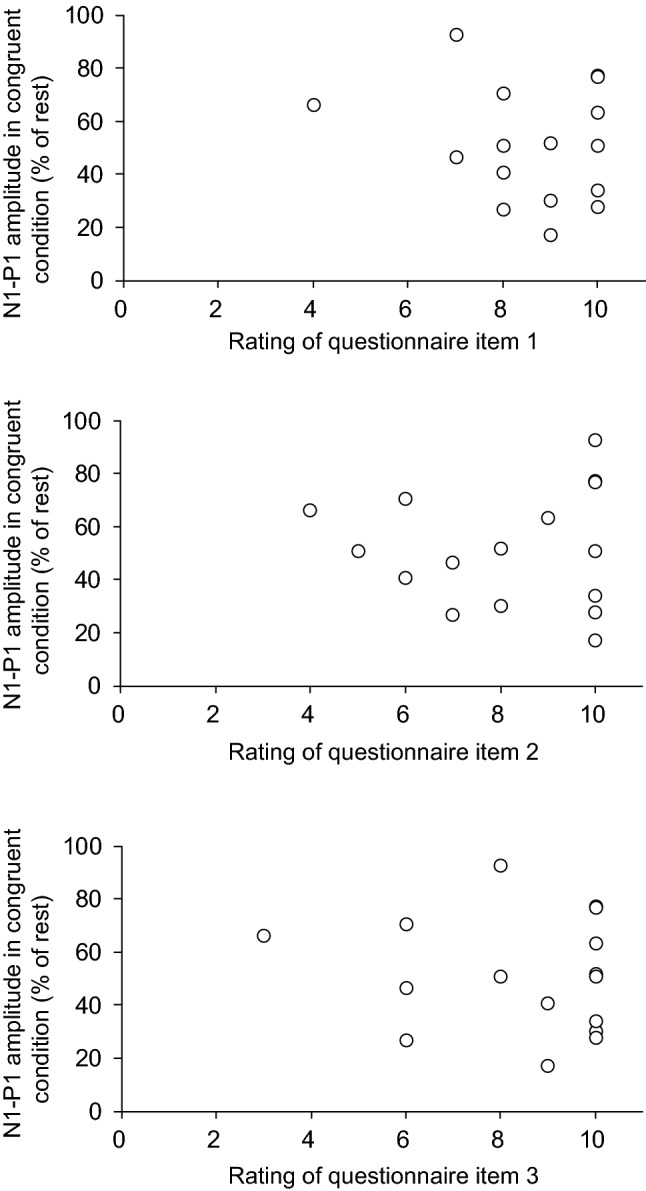
Correlation between the ratings of questionnaire items 1, 2, and 3 and the N1-P1 amplitudes in the congruent condition. Values on the ordinate indicate the N1-P1 sizes in the congruent condition as a percentage of those obtained from the rest.

Questionnaire items 1, 2, and 3 showed high ratings in the congruent stroking condition (Fig. 4). Therefore, these questionnaire items were considered to be related to changes in the sense of body ownership. To calculate the degree of decrease in N1-P1 amplitude during RHI, the amplitude in the congruent stroking condition was normalised with respect to that obtained in the rest condition. We compared the degree of decrease in the N1-P1 amplitude in the congruent stroking condition with ratings of questionnaire items 1, 2, and 3 by utilising the Spearman rank correlation coefficient (Fig. 5). There were no significant correlations between them (questionnaire item 1: ρ = -0.093, *p* = 0.73; questionnaire item 2: ρ = 0, *p* = 1; questionnaire item 3: ρ = − 0.041, *p* = 0.88).

### Experiment 2

**Figure 6:**
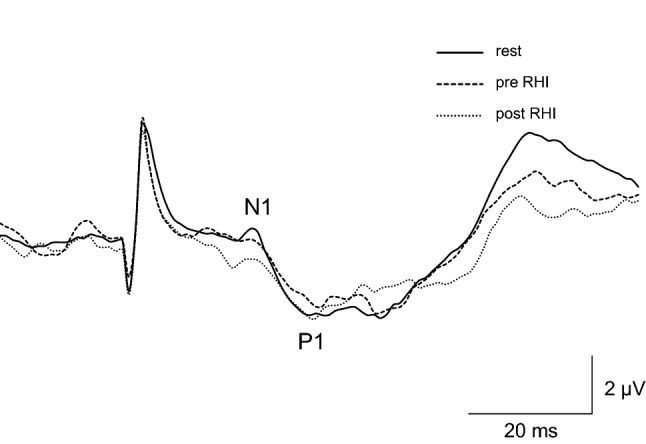
Grand averaged waveforms of somatosensory evoked potentials (SEPs) during all conditions in experiment 2.

Figure 6 shows the grand averaged waveforms of SEP in the rest condition, and during the pre- and post-RHI periods. It can be seen that the N1-P1 component showed an attenuation in amplitude during the pre-and post-RHI periods. The averages for SEP in the rest condition, and during the pre- and post-RHI periods were 28.7 ± 8.3, 26.7 ± 4.0, 27.1 ± 7.3, respectively.

**Figure 7:**
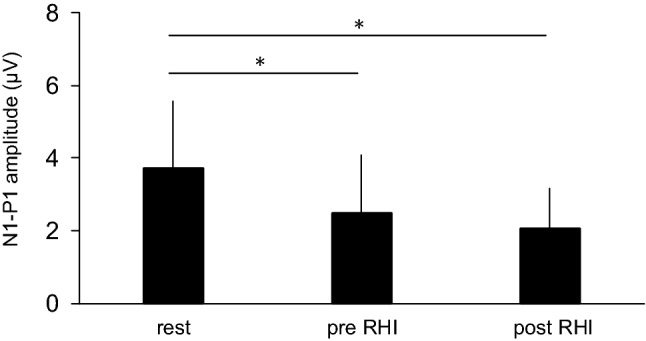
Group means of N1-P1 amplitudes during the rest, pre rubber hand illusion (RHI), and post RHI conditions in Experiment 2. Data are represented as mean ± one SD. *p < 0.05.

The amplitudes of the N1-P1 components in all conditions are shown in Fig. 7. One-way repeated measures ANOVA revealed a significant main effect for the conditions (F(2,26) = 8.39, *p* = 0.002, η^2^ = 0.39). Post-hoc comparisons demonstrated that the N1-P1 component during both the pre-and post-RHI periods was significantly smaller than that in the rest condition (pre RHI: *p* = 0.037, post RHI: *p* = 0.012). There was no significant difference in the N1-P1 amplitudes between the pre- and post-RHI periods (*p* = 0.74).

## Discussion

In this study, we investigated the modulation of SEPs during the RHI. Compared with previous studies that showed attenuation of SEP components approximately 50 ms after stroking during the RHI^13,21^, our data indicates that the earliest component of SEPs, N1-P1 response, was attenuated when RHI was elicited. However, the degree of decrease in the N1-P1 amplitude in the congruent stroking condition was not significantly correlated with the subjective ratings of the questionnaire. This suggests that the attenuation of the N1-P1 amplitude is not a direct cause of the occurrence of the RHI. Considering that the N1-P1 amplitude attenuated before the participants felt the rubber hand as their own, the attenuated sensory processing in the primary somatosensory cortex during the RHI seems to be partially involved in the occurrence of the illusion.

SEPs have been typically recorded by continuous electrical stimulation with short interstimulus intervals (1–2 Hz)^28–33^. In this study, the intensity of the electrical stimulation applied to the median nerve was adjusted to produce a slight twitch of the abductor pollicis brevis muscle. If continuous electrical stimulation was provided, a continuous twitch of the thumb occurred in the participants. This ensured that the participants always felt the movements of their hidden real hand. This condition interrupted the occurrence of an RHI. Therefore, in this study, the interstimulus interval of electrical stimulation was adjusted by the experimenter (around 10 s) and the frequency did not differ among the four conditions (Fig. 3). Although the modulation of the SEP amplitude depends on the frequency of the electrical stimulation^34^, it was not the cause of the modulations of the N1-P1 amplitude in this study. However, the long interstimulus interval does not ensure a large number of waveforms triggered by electrical stimulation as the experiment time increases and the physical or psychological load of the participants increases accordingly. Thus, we adopted a special arrangement of recording electrodes, introduced by Brooke et al.^35,36^. A merit of this arrangement was that it recorded a clear waveform of the early components of SEP with a small sample size.

Previous studies used brush-stroke stimulation to evoke SEPs during RHI^13,16–18,21^. The obtained SEPs were derived from tactile stimulation that is necessary to produce the RHI and involved some components that were thought to originate from the primary somatosensory cortex^19,20^. However, the merits of brush-stroke-evoked SEPs do not include the recognisable earliest component of the primary somatosensory cortex that is observed around 20 ms after stimulation^22–25^. The earliest component was generated from Brodmann area 3b^26,27,37^. This was also confirmed in SEPs recorded at the cortical surface during neurosurgery^38^. Thus, the earliest component of SEP, the N1-P1 component, was thought to reflect the initial sensory processing in the primary somatosensory cortex. Electrical stimulation was used to obtain the earliest components. One might think that inflow of somatosensory inputs induced by electrical stimulation to the cerebral cortex does not originally occur during procedures in the RHI. Although somatosensory inflow produced by electrical stimulation is unnatural, we believe that modulation of the N1-P1 amplitude is the optimal index for evaluating the initial somatosensory processing in the primary somatosensory cortex^35,36^.

There is no consensus on the role of the primary somatosensory cortex in the occurrence of the RHI. Previous neuroimaging studies have demonstrated neural correlates of the RHI in the premotor and parietal cortices^4,7,8^. A similar finding was reported with ECoG in patients^11^. Furthermore, Rao and Kayser^12^ used EEG and demonstrated that neurophysiological correlates of the RHI were observed in electrodes of frontocentral areas. In those studies, no neural correlates of the RHI were observed in the primary somatosensory cortex. In contrast to these findings, some recent studies suggested modulation of sensory processing in the primary somatosensory cortex during RHI. Zeller et al.^13^ reported that brush-evoked SEP responses of approximately 50 ms were attenuated during RHI. The component is thought to originate from the primary somatosensory cortex, although it does not reflect the initial stage of sensory processing in the area^19,20^. More direct evidence has shown that neural activity in the somatosensory cortex of monkeys changed during RHI^15^. In addition, a transcranial magnetic stimulation study indicated that functional inhibitory connections from the primary somatosensory cortex to the primary motor cortex were reduced during RHI^14^. This suggests reduced tactile somatosensory processing in the primary somatosensory cortex during RHI. Our findings support these reports and suggest that the primary somatosensory cortex is partly involved in the occurrence of the RHI. Since discrepancies in findings are likely to depend on differences in the methods used, further research is required considering the experimental conditions.

The transmission of somatosensory signals to the primary somatosensory cortex is diminished during active or passive movements and tactile stimulation of the hand^29,30,39–43^. This mechanism is called “gating” The gain in the SEP amplitude is modulated by centrifugal and centripetal gating mechanisms^30^. The former is that the efferent signals induced by the motor command from the motor-related areas suppress the ascending somatosensory signals. The latter is interfering effects between the given sensory afferent signals induced by electrical stimulation of the nerve and the afferent feedback from the skin caused by tactile stimulation of the hand. In this study, we did not examine the participants’ muscular activities using electromyography. However, we do not consider muscle contractions as the main cause of the modulation of N1-P1 amplitude in this study. This is because we asked the participants to relax throughout the experiment, and the four experimental conditions were performed in a random order. It is unlikely that the decrease in N1-P1 amplitude in the congruent stroking condition was caused only by the muscle contractions of the participants. In addition, we believe that the decrease in the N1-P1 amplitude during RHI is not explained only by traditional centripetal gating mechanisms. In Experiment 1, the N1-P1 amplitude in the tactile stimulation condition was substantially smaller than that in the rest condition. This finding would be affected by centripetal gating^29^. Similar tactile stimulations were provided to the participants in both the congruent and incongruent stroking conditions. Despite the same manner of the tactile stimulation, N1-P1 amplitude in the congruent stroking condition was significantly attenuated compared with those in the incongruent and tactile stroking conditions (Fig. 2). Thus, attenuation of the N1-P1 amplitude during RHI would not be caused by only centripetal gating. We do not know the mechanisms for this modulation, but certain mechanisms related to the occurrence of the illusion might centrally affect the modulation of the transmission of somatosensory signals.

The subjective ratings for the questionnaire are typically used as an index for evaluating changes in the sense of body ownership^3^. In this study, ratings of questionnaire items 1, 2, and 3 in the congruent stroking condition were high, which is consistent with previous studies^3,44–46^. This indicates that our procedure properly induced the RHI. The degree of decrease in the N1-P1 amplitude in the congruent stroking condition was not significantly correlated with subjective ratings of the questionnaire (Fig. 5). We infer that attenuation of the N1-P1 amplitude is not a direct cause of the occurrence of the RHI. Rather, the modulation of the N1-P1 amplitude seems to be partially involved in the occurrence of the illusion.

In Experiment 1, the electrical stimulation was continuously provided at intervals of approximately 10 s. In this case, the SEP waveform might be obtained from the electrical stimulation given before and after the occurrence of the illusion. Therefore, the findings of the experiment were not able to explain whether the occurrence of the RHI is followed by modulations of somatosensory processing in the primary somatosensory cortex, or the modulation of somatosensory processing occurs before the occurrence of the illusion. To address this, we performed Experiment 2 and found that the N1-P1 amplitude was attenuated before the occurrence of the illusion. According to a model of body ownership during RHI^5,47^, the posterior parietal cortex integrates visual and somatosensory information of touch before the occurrence of the RHI. This has also been clarified using intracranial electrodes implanted in patients^11^. When synchronised visual capture of the rubber hand being touched and tactile inputs from the occluded hand are integrated, the posterior parietal cortex is thought to be involved in the resolution of the conflict between visual and tactile information^5,47^. Makin et al. ^5^ mentioned that during RHI, the integration of sensory information is weighed heavily in favour of vision. This is likely to lead to a reduction in the weight of somatosensory inputs^5,48^. This idea was partially supported by behavioural^49,50^ and neurophysiological^14^ studies. The findings of this study, which showed that the N1-P1 amplitude is attenuated during RHI, would explain this idea. Considering the findings of Experiment 2, although the direct cause of the RHI would be multisensory integration in the parietal cortex, somatosensory processing may need to be centrally gated in the primary somatosensory cortex.

This study has two limitations. First, we cannot distinguish whether the N1-P1 response originates from cutaneous afferent or muscle afferents. Modulation of cutaneous afferent inputs is meaningful in the RHI paradigm. The median nerve is a mixed nerve containing both muscle and cutaneous afferents, implying that any changes in the N1-P1 amplitude may not be solely attributable to only one of these groups of afferents. Gandevia and Burke^51^ demonstrated an intramuscular and percutaneous mixed nerve trunk stimulating technique, in which muscle afferents contributed to the recorded N1-P1 potential. In this study, we did not record N1-P1 potentials elicited by stimulation of only cutaneous afferents (e.g. stimulating the nerve of the digit). This is because the responses obtained by the stimulation of the digit nerve are small and require a large number of sample sizes^52,53^. Therefore, this study suggests that somatosensory signals to the brain are diminished during RHI, although the modality of afferents is not specified. Second, we did not provide electrical stimulation while participants looked at their real hand being stroked. If the RHI produces the embodiment of the rubber hand, the gating of somatosensory processing might be similar to that in the condition where the participants visualize their hand being stroked. This suggests that sensory gating during the RHI may be a physiological signature of the embodiment of the rubber hand. Further studies are needed to solve these problems.

In summary, our results suggest that attenuation of somatosensory processing occurs at the primary somatosensory cortex during RHI. Furthermore, the attenuation starts before the occurrence of the illusion. In addition to the fact that multisensory integration in the parietal cortex is thought to be a direct cause of the occurrence of the illusion, we consider that attenuation of somatosensory processing at the entrance of the cerebral cortex does contribute to the occurrence of changes in feelings of limb ownership. This study has gone some way toward enhancing our understanding of the neural mechanisms underlying the occurrence of the RHI.

## Methods

### Participants

Thirty male volunteers aged 20 to 24 years, naïve to the purpose of the experiments, participated in this study. Sixteen participants participated in Experiment 1, and the remaining 14 participated in Experiment 2. All participants had normal findings on physical and neurological examinations and provided written informed consent. This study was approved by the Human Research Ethics Committee of the Faculty of Education, Kumamoto University. The experiments were conducted in accordance with the Declaration of Helsinki.

### Recording

Electroencephalographic (EEG) signals for determining SEP were recorded from C4′ (2 cm behind C4) referenced to Fpz’ (2 cm caudal to Fpz) in accordance with the international 10–20 system. This arrangement of recording electrodes was introduced by Brooke et al.^35^, and was reported to successfully determine SEP during pedaling^54–56^ and sustained finger muscle contraction with fatigue^53^. Vertical and horizontal electrooculograms (EOGs) were also recorded above and below the right orbital fossa. The EEG and EOG signals were amplified and filtered at a band-pass of 5–100 Hz and 0.5–120 Hz, respectively. All data were stored on a hard disk with a sampling rate of 1 kHz.

### Electrical stimulation

The left median nerve was stimulated on the palm side of the wrist with surface Ag/AgCl disk electrodes (Ø 1.5 cm). The cathode was placed 2 cm proximal to the anode. The electrode was fixed on the median nerve so as not to move during recording. Constant current square wave pulses (duration, 0.2 ms) were provided, and the intensity was adjusted to produce a slight twitch of the abductor pollicis brevis muscle. The inter-stimulus interval was approximately 10 s, which was controlled by the experimenter (see below). This was intended to prevent the participants from anticipating the timing of the electrical stimulation.

### Experiment 1

The experiment was conducted in a dimly lit room. Participants sat on a chair throughout the experiment. Both the participants’ left hand and the fake left hand (see below) wore identical light blue coloured rubber gloves to eliminate differences in appearance between them^6^. Participants put their left hand and forearm inside a wooden frame with the forearm in the prone position. A fake left hand constructed of rubber was placed in a prone position 19 cm medial to the participants’ unseen left hand. The experimenter put black clothes on both the left upper arm of the participants and the forearm of the fake hand. Therefore, participants were able to see only the fake hand throughout the experiment.

Electrical stimulation was applied to the participants’ left median nerve under one of the following four conditions:

*The congruent stroking condition*: The experimenter asked the participants to view the fake hand, and delivered tactile stimulation for 4 min with the use of two identical paintbrushes. At this time, both the participant’s hand and the fake hand were stroked simultaneously and at the same location. During the congruent tactile stimulation, the experimenter applied electrical stimulation by pressing the foot switch at his foot. The interstimulus interval of the electrical stimulation was approximately 10 s, which was adjusted by the experimenter.

*The incongruent stroking condition*: The timing and location of stroking did not match between the participant’s hand and the fake hand. Other procedures were the same as those in the congruent stroking condition.

*The tactile stimulation condition*: The fake hand was removed and a small cube with a side of 1 cm was placed where the fake hand was. The experimenter asked the participants to view the cube, and provided the tactile stimulation to the participants’ left hand for 4 min. During the tactile stimulation, the experimenter applied electrical stimulation in a manner similar to that of the congruent stroking condition.

*Rest condition*: The participants viewed the small cube that was the same as the used in the tactile simulation condition. Tactile stimulation was not provided to the participants’ left hand for 4 min. However, only the electrical stimulation was applied to the participants’ median nerve in a similar manner to that of the congruent stroking condition.

The four experimental conditions were repeated twice each in a random order. Between each condition, there was a resting period of 5 min.

After completing each condition, participants were also asked to answer the RHI questionnaire. The questionnaire consisted of eight statements that were adopted from Botvinick and Cohen’s^3^ original report. The questions were as follows: (Q1) it seemed as if I were feeling the touch of the paintbrush in the location where I saw the rubber hand touched, (Q2) it seemed as though the touch I felt was caused by the paintbrush touching the rubber hand, (Q3) I felt as if the rubber hand were my hand, (Q4) it felt as if my (real) hand were drifting towards the rubber hand, (Q5) it seemed as if I might have more than one left/right hand or arm, (Q6) it seemed as if the touch I was feeling came from somewhere between my own hand and the rubber hand, (Q7) it felt as if my (real) hand were turning ‘rubbery’, (Q8) it appeared (visually) as if the rubber hand were drifting towards my hand. The participants responded by choosing a value on a 10-point scale ranging from 1 to 10, with 1 corresponding to ‘strongly disagree’ and 10 to ‘strongly agree’.

### Experiment 2

In this experiment, only the rest condition and the congruent stroking condition in Experiment 1 were performed. In the congruent stroking condition, the experimenter provided both tactile and electrical stimulations, as in Experiment 1. The participants were asked to press a button that was put under their right hand with the right hand when they felt the rubber hand as their own. This allowed the experimenter to know the timing of occurrence of the RHI^57^. The experimenter stopped the synchronous tactile stimulation about 20 s after the participants experienced the RHI, that is, after pressing the button. This procedure was repeated 40–50 times with a 10–20 s break. Other procedures were the same as those performed in Experiment 1. The two experimental conditions were conducted in random order.

### Data analysis and statistics

In an offline analysis, measurement of the SEP amplitude was taken from N1 (first negative peak about 20 ms after electrical stimulation) to P1 (first positive peak about 25 ms) at the C4′ location over the scalp. Variations in EOG signals greater than 80 μV were excluded from SEP averaging.

In the experiment 2, to confirm time course modulation of N1-P1 amplitude, we defined the period from 10 s before the participants pressed the button to the time the button was pressed as “pre-RHI”. This is because RHI occurs 11.3 ± 7.0 s after tactile stimulation^4^. We also defined the period from when the participants pressed the button to 10 s later as “post-RHI”. SEPs were separately averaged over the two periods.

To modulate the N1-P1 amplitude and the frequency of the electrical stimulation across the experimental conditions, a one-way repeated measures analysis of variance (ANOVA) was performed. To analyse the assumption of sphericity prior to the repeated measures ANOVA, we used Mauchly’s test of sphericity. If the result of the test was significant and the assumption of sphericity was violated, the Greenhouse–Geisser adjustment was used to correct for the sphericity by altering the degrees of freedom using a correction coefficient epsilon. For post-hoc comparisons, multiple pairwise tests with Bonferroni’s correction were performed. In Experiment 1, to calculate the degree of changes in N1-P1 amplitude during RHI, the amplitude in the congruent stroking condition was normalised with respect to that obtained in the rest condition. The relationships between the obtained N1-P1 amplitude (% of rest condition) and ratings of the questionnaires were tested using the Spearman rank correlation coefficient. Data are expressed as mean ± standard deviation. Significance was set at *p* < 0.05. IBM SPSS Statistics was used for all statistical analyses.
